# Insight into the Lytic Functions of the Lactococcal Prophage TP712

**DOI:** 10.3390/v11100881

**Published:** 2019-09-20

**Authors:** Susana Escobedo, Ana Belén Campelo, Udo Wegmann, Pilar García, Ana Rodríguez, Beatriz Martínez

**Affiliations:** 1 Dairy Safe group, Instituto de Productos Lácteos de Asturias (IPLA), Consejo Superior de Investigaciones Científicas (CSIC), 28014 Madrid, Spain; 2 School of Chemistry, University of East Anglia, Norwich Research Park, NR4 7TJ Norwich, UK

**Keywords:** bacteriophage, endolysin, *Lactococcus*, cell wall, *O*-acetylation

## Abstract

The lytic cassette of *Lactococcus lactis* prophage TP712 contains a putative membrane protein of unknown function (Orf54), a holin (Orf55), and a modular endolysin with a N-terminal glycoside hydrolase (GH_25) catalytic domain and two C-terminal LysM domains (Orf56, LysTP712). In this work, we aimed to study the mode of action of the endolysin LysTP712. Inducible expression of the holin-endolysin genes seriously impaired growth. The growth of lactococcal cells overproducing the endolysin LysTP712 alone was only inhibited upon the dissipation of the proton motive force by the pore-forming bacteriocin nisin. Processing of a 26-residues signal peptide is required for LysTP712 activation, since a truncated version without the signal peptide did not impair growth after membrane depolarization. Moreover, only the mature enzyme displayed lytic activity in zymograms, while no lytic bands were observed after treatment with the Sec inhibitor sodium azide. LysTP712 might belong to the growing family of multimeric endolysins. A C-terminal fragment was detected during the purification of LysTP712. It is likely to be synthesized from an alternative internal translational start site located upstream of the cell wall binding domain in the lysin gene. Fractions containing this fragment exhibited enhanced activity against lactococcal cells. However, under our experimental conditions, improved in vitro inhibitory activity of the enzyme was not observed upon the supplementation of additional cell wall binding domains in. Finally, our data pointed out that changes in the lactococcal cell wall, such as the degree of peptidoglycan *O*-acetylation, might hinder the activity of LysTP712. LysTP712 is the first secretory endolysin from a lactococcal phage described so far. The results also revealed how the activity of LysTP712 might be counteracted by modifications of the bacterial peptidoglycan, providing guidelines to exploit the biotechnological potential of phage endolysins within industrially relevant *lactococci* and, by extension, other bacteria.

## 1. Introduction

Bacteriophages use two different mechanisms to lyse host cells, while some ssDNA and RNA viruses inhibit peptidoglycan synthesis, dsDNA viruses display an endolysin-mediated breakdown of peptidoglycan (PG) caused by the enzymatic degradation of the PG of the host bacterium by phage-encoded hydrolases, i.e., endolysins produced at the end of their lytic multiplication cycle [1]. Endolysins must traverse the plasma membrane in order to reach their cell wall target. As recently reviewed by [2], one of the most common and well-studied mechanisms is the holin–endolysin cell lysis system or canonical model. Holins are small transmembrane proteins that, upon oligomerization, form holes in the cytoplasmic membrane of bacteria allowing for the passive diffusion of endolysins to the cell wall. In recent years, non-canonical lysis systems have been described where endolysins are exported to the cell wall (CW) without passing through a holin pore in the plasma membrane. For several bacteriophages, the endolysin is produced with a non-cleavable N-terminal type II signal anchor instead, where it stays embedded in the inner cell membrane in an inactive form (SAR system). Membrane depolarization that is caused by the holin is required to release the SAR anchor, so the endolysin can fold into its active conformation and get access to the cell wall [3]. In other examples, endolysin export was shown to depend on the host’s general secretion pathway (the Sec system). These Sec-dependent endolysins have been described for phages fOg44 from *Oenococcus oeni* and *Lactobacillus plantarum* phage Øg1e. They produce endolysins that are endowed with a typical Sec-type signal peptide [4,5]. As an alternative to the Sec pathway, endolysins might bind precursors of CW secondary polymers in the cytoplasm and be co-transported to the CW. This seems to be the case for the endolysin of *Streptococcus pneumoniae* phage SV1, whose translocation to the CW was proposed to be coupled to the synthesis and transport of choline-containing teichoic acids [6]. On the other hand, the secretion of the endolysin of mycobacteriophage Ms6 depends on the activity of a phage-encoded chaperone [7,8].

Several studies have shown that endolysins remain in an inactive form after translocation to the cell wall, and various processes are needed to either activate them or enhance their catalytic activity [2]. One of the most common modes of endolysin activation is the depolarization of the cytoplasmic membrane through the activity of holins or pinholins (small holins). This explains why many phages still produce holins during their infection cycle, although their endolysin can be translocated in a holin-independent manner [9]. Indeed, the addition of a membrane disrupting agent, such as the pore-forming bacteriocin nisin along with the purified endolysin from phage fOg44, was necessary to trigger lysis of *Oenococcus oeni cells* [10], while nisin also enhanced the lytic activity of the *Staphylococcus* endolysin LysH5 [11]. The majority of highly active lysins have a multi-domain architecture that is composed of separate catalytic (CD) and cell wall binding domains (CBD) [12,13,14]. CBDs specifically bind to the host CW and confer specificity of the endolysin for certain CW types. Various conserved binding modules have been described in the literature. The LysM domain is reportedly the most common domain in PG hydrolases and it is known to bind to *N*-acetyl-glucosamine residues in the sugar backbone of the PG [15]. With the exception of the multimeric endolysin PlyC, whose subunits have separate genes [16] all other endolysins studied so far are encoded by a single gene and seem to be monomeric when purified. Endolysin genes that encode the expected full length and truncated products through alternative translation initiation have also been described. The enterococcal phage F170/08 endolysin Lys170 is an example of an endolysin, whose lytic activity depends on the assembly of additional CBD subunits, arising from an in-frame alternative start codon, with the full-length monomer [17], while in the case of endolysin CTP1L, which targets *Clostridium tyrobutyricum*, CBDs appear to modulate endolysin activity, at least in vitro [18].

In this work, we aimed at studying the lytic functions of the lactococcal temperate phage TP712. This phage belongs to the P335 group of the Siphoviridae family of phages, which infect *Lactococcus lactis*, a bacterium widely used as a starter for the manufacture of fermented dairy products, such as cheese and sour cream. Specifically, this prophage (t712, in [19] and φTP712 in [20]) is integrated in the chromosome of the laboratory workhorse *L. lactis* MG1363 and derivatives thereof. TP712 appears to be sensitive to host mutations and productive infections do not occur in *L. lactis* Δ*ftsH*, lacking the housekeeping and stress-responsive membrane protease FtsH [21,22]. In this work, our results showed that: (i) dissipating the proton motive force is required to relieve the mechanisms that restrain endolysin activity, (ii) LysTP712 is a secreted endolysin whose signal peptide must be removed in a Sec-dependent manner to be active, and (iii) the in vitro activity of LysTP712 is hindered by an altered PG composition.

## 2. Materials and Methods

### 2.1. Bacterial Strains and Growth Conditions

Table 1 lists the microorganisms used in this work. *Lactococcus lactis* strains were grown in M17 (Oxoid, Madrid, Spain) supplemented with 0.5% glucose (GM17) at 30 °C. When needed, chloramphenicol (10 μg/mL) was added to the culture medium for plasmid selection. For expressing genes under the control of the nisin promoter, 2 ng/mL of purified nisin (a gift from Applin&Barret, UK) was added and the growth curves were followed in a microtiter plate reader Benchmark Plus Microplate Spectrophotometer (BioRad, Hercules, CA, USA). *E. coli* was grown in Luria-Bertani broth [23] at 37 °C with agitation. The plasmids were selected on the same medium supplemented with ampicillin (100 μg/mL).

### 2.2. General Molecular Techniques

Chromosomal DNA was isolated with the GenElute Bacterial Genomic DNA Kit (Sigma, North Watford, UK). Plasmids were purified while using the High Pure Plasmid Isolation Kit (Roche, Basel, Switherland). DNA fragments were isolated using the Gel Band Purification Kit (GE Healthcare, Chicago, IL, USA). All of the restriction endonucleases were purchased from EURx (Gdańsk, Poland) and Takara (Kusatsu shi, Japan). Ligations of the fragments into the cloning vectors were carried out with T4 ligase (Fisher Scientific, Waltham, MA, USA) at a final volume of 20 μL; reactions were incubated for 14 h at 16 °C. All the constructions were checked by DNA sequencing (Macrogen, Madrid, Spain).

### 2.3. Plasmids Construction

Table 2 describes the expression plasmids. They were constructed by amplifying the desired genes by the polymerase chain reaction (PCR) while using Phusion DNA polymerase (Fisher Scientific) and cloning under the nisin inducible promoter (PnisA) into *L. lactis* plasmid pUK200 or into the *E. coli* vector pET21a. The genes were amplified while using 10 ng of chromosomal DNA of *L. lactis* UKLc10 TP712 as template.

To construct plasmid pUK200_AH_H_L, a 1639 bp fragment carrying holin and lysin genes of TP712 was PCR amplified using TP712 genomic DNA and primers holin_f (5′ ACGGCTTACAAATTGTCAGTGG3′) and lysin reverse (5′ AACACCCGCCGAAGCGGG 3′) and subsequently cloned into SmaI restricted pUK200, resulting in pUK200_AH_H_L.

To yield the plasmid pLysTP712 (Table 2), primers Lys TP-64-F (5′ AAAAAAGTAATTAAAAAAGCTGCCATTGG 3′) and LysTP-6xHis-Stop-R (5′ GCAGGATCCTCAGTGATGGTGATGGTGATGATAATTTAAAGTTTGACCAGCATAAATCAAATTAGG3′) at a final concentration of 0.3 μM were used. The product was digested with BamHI and ligated into pUK200 previously digested with NcoI, Klenow filled (0.04 mM of each dNTP and 3.5 units of Klenow enzyme in a final volume of 100 μL) and then digested with BamHI. A 5′-truncated version of *lys*TP712 was also cloned in pUK200 to make pΔSPLysTP712 following a similar strategy. The primers for PCR were LysFi (5′ ACTGGATCCATGGCAGTCGGTG) and LysR (5′ ACTGACTGGATCCTTAATAATTTAAAGTTTG 3′). The PCR product and the vector pUK200 were both digested with NcoI and BamHI.

To generate plasmid pETLysTP712 we used primers LysNdeI-F (5′ ACTGACTGCATATGGCAGTCGGTGAC) and LysXhoI (5´ ACTGACTGCTCGAGATAATTTAAAGTTTG 3′). The PCR fragments that were obtained were digested with NdeI and XhoI and ligated to pET21a vector cut with the same restriction enzymes. His-tagging of the endolysin CBD was made while using primers LysMmet1 (5′ ACTGACTGCATATGACGGTTAAAGTAA 3′) and Lys XhoI. The PCR product was digested with NdeI and XhoI and cloned into pET21a to generate pETLysTP712CBD (Table 2).

### 2.4. Affinity Purification of Recombinant LysTP712

To study the enzymatic activity of TP712 endolysin, LysTP712 that was endowed with a C-terminal hexahistidine tail was overproduced in *L. lactis* and *E. coli* hosts. *L. lactis* plysTP712 were induced with nisin (2 ng/mL) at an OD_600_ of 0.5 and grown at 30 °C for 2 h. *E. coli* pETlysTP712 were also induced at OD_600_ of 0.5 but with IPTG (100 µg/mL) and grown at 37 °C for 2 h. The cells were then harvested by centrifugation (6000× *g*, 15 min., 4 °C), resuspended in 50 mM sodium phosphate buffer (pH 6.8), and disrupted (twice, 2.5 Kbar) in a One Shot Cell Disrupter (Constant Systems LTD, Daventry, UK). Next, the cell lysates were centrifuged at 10,000× *g* for 15 min. and the resulting pellet was dissolved in 100 mM NaH_2_PO_4_, 10 mM Tris·HCl, 8 M urea, pH 8. After centrifugation, the solubilized protein was purified under denaturing conditions by His tag affinity chromatography while using Ni-NTA Suplerflow Resin (Qiagen, Hilden, Germany). Following the manufacturer’s recommendations, after loading the samples, the resin was washed with the same buffer but at pH 6.3 and, subsequently, two fractions were eluted at two different pH values: pH 5.9 (fraction I-LysTP712FL) and pH 4.5 (fraction II-LysTP712). The protein concentration was determined by the Bradford assay (Bio-Rad Laboratories, Hercules, CA, USA) while using bovine serum albumin as a standard.

Sodium dodecyl sulfate (SDS)-polyacrylamide gel electrophoresis was performed, as described by [28], in a BioRad Mini-Protean gel apparatus (BioRad). For zymograms, 0.2% autoclaved and lyophilized *L. lactis* NZ9000 cells were included in 16% polyacrylamide gels for the detection of bacteriolytic activity, as previously described [29]. Briefly, after electrophoresis, the gels were washed three times with milliQ H_2_O for 10 min. at room temperature and incubated overnight at 30 °C with gentle shaking in 50 mM MES-NaOH pH 6.0 and 0.1% triton X-100. The gels were then rinsed with distilled water. Prestained molecular weight standards were electrophoresed on the same gel to estimate molecular weight.

### 2.5. N-Terminal Sequence Analysis

Electrophoresis of purified protein fractions was performed at 100 V for 1 h in a 16% SDS-PAGE gel before transfer of the proteins onto a polyvinylidene difluoride membrane (Bio-Rad). N-terminal sequence determination was performed by Edman degradation analysis on an Applied Biosystems Procise 494 protein sequencer at the CIB Protein Chemistry Facility (Spain).

### 2.6. LysTP712 Activity Assays

The activity of the endolysin LysTP712 when synthesized within the host (lysis from within) was determined in mid-log-phase cultures (OD_600_, 0.4) of *L. lactis* NZ9000 carrying pLysTP712, p∆SPLysTP712, or the control plasmid pUK200. The cells were induced with 2 ng/mL nisin and incubated for two more hours before adding 0, 12.5 (3.75 nM), 25 (7 nM), or 50 ng/mL (15 nM) of nisin. After nisin treatment, the OD_600_ of the suspensions over time was monitored in a microtiter plate reader. Growth inhibition was calculated after 2 and 16 h after treatment, relative to each induced culture to which no nisin was added. The differences in growth were compared to that of the empty plasmid control *L. lactis* pUK200 with a two-tailed Student’s test and significance was set at a *p*-value threshold of 0.05.

The two purification fractions (fraction I-LysTP712FL and fraction II-LysTP712) were first diluted 100-fold in 50 mM Na_2_HPO_4_/NaH_2_PO_4_ buffer, pH 6.8, to dilute urea down to 80 mM and immediately used for the activity assays to determine the activity of the recombinant LysTP712 when added from the outside (lysis from without). Aliquots of 100 µL were serially diluted in 50 mM Na_2_HPO_4_/NaH_2_PO_4_ buffer with 80 mM urea and mixed with 100 µL of *L. lactis* MG1363 cultures (OD_600_, 0.4) growing in GM17 broth. These assays were performed in a microtiter plate and the OD_600_ was periodically measured. Final protein concentrations in the wells were 5, 2.5, 1.25, 0.6, and 0.3 µg/mL. The cells mixed with buffer were only used as control. To evaluate the activity of LysTP712FL (fraction I) and CBD mixtures, fraction I and purified LysTP712CDB were co-incubated for 20 min. at 30 °C in 50 mM Na_2_HPO_4_/NaH_2_PO_4_, 80 mM urea, pH 6.8, with molar ratios (LysTP712FL:LysCBD) of 1:0, 1:1, 1:3 and 1:5. The pre-incubation of cells with fixed concentrations of CBD 0.5, 0.3, 0.15, and 0 µM to get molar ratios of 1:10, 1:6, 1:3, and 1:0 with LysTP712FL (0.05 µM) endolysin was also assayed. The final urea concentration in all these assays was 40 mM, which did not inhibit *L. lactis* MG1363 growth and avoided protein precipitation. For the comparison of the susceptibility to LysTP712 of *L. lactis* VES3902 and *L. lactis* VES4289, activity assays were carried out, as described above, in a microtiter plate and incubating cells with 2.5 µg/mL of recombinant LysTP712 (Fraction II).

Growth data (OD_600_) from replicates of the lysis from without experiments was taken after 120 min. of treatment and was analyzed with a two-tailed Student´s *t*-test. A *p*-value threshold of 0.05 was set for significance.

### 2.7. Phage DNA Sequence Analysis

The ProtParam tool [30] was used to determine theoretical molecular weight and isoelectric points of the deduced phage proteins. Signal peptide and cleavage site was predicted by online SignalP 5.1 Server [31]. Amino acid sequence homology analysis and the conserved domains were based on BLASTp program [32] and Pfam searches [33]. Promoter predictions were done with Bprom [34].

## 3. Results

### 3.1. Expression of the TP712 Holin and Endolysin Genes Hampers Growth of L. lactis

**Figure 1:**
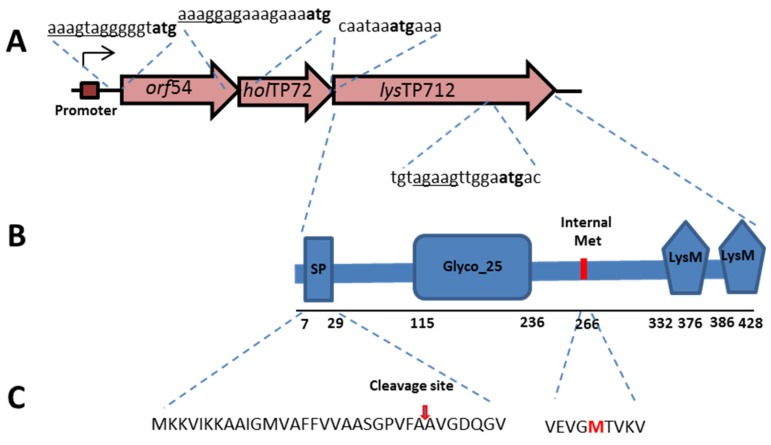
Features of the TP712 lytic cassette (GenBank AAX13238.1). (**A**) The lytic cassette of TP712 contains a gene of unknown function (*orf54*), a putative holin gene *holTP712* (*orf55*) and the lysin gene lysTP712 (*orf56*); start codons bold, RBS underlined. (**B**) Protein domains identified in the TP712 endolysin encoding gene and position of a putative internal translational start site (Met). SP, cleavable signal peptide; Glyco_25, glycosyl hydrolase catalytic domain, and two LysM cell binding domains. (**C**) N-terminal signal peptide amino acid sequence. Cleavage-site is indicated by an arrow.

According to the TP712 genome sequence (GenBank AY766464), the lytic cassette of TP712 comprises a holin gene *holTP712 (orf55)*, from which two small membrane proteins are synthesized by virtue of an alternative start codon, and a downstream lysin gene *lysTP712 (orf56)*, coding for a modular endolysin with a glycosil hydrolase GH_25 catalytic domain (CD) and a 2× LysM cell wall binding domain (CBD). These two orfs match the holin ORF 850 and the endolysin ORF 851 described in the prophage analysis of *L. lactis* MG1363 by Ventura et al. [19]. Upstream of these genes, there is another small *orf54* that is preceded by a ribosomal binding site (AAAGTAGG). Closer inspection of the DNA sequence revealed the presence of a putative promoter ATAACA-19 nt-TTTTAATA upstream of *orf54* (Figure 1). Moreover, lysTP712 likely encodes for a pre-endolysin with an N-terminal leader peptide that might be cleavable or not [22].

**Figure 2:**
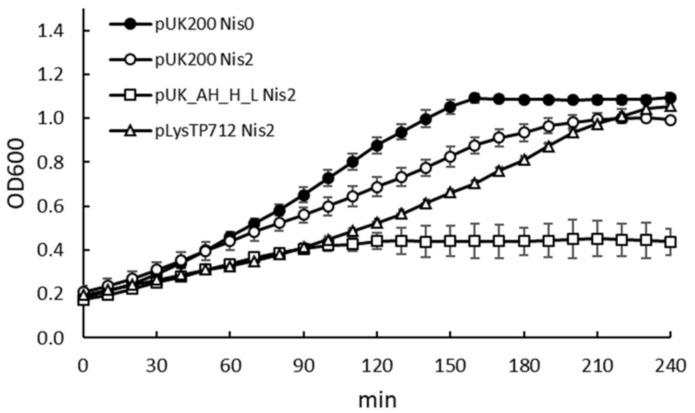
Growth of *L. lactis* NZ9000 expressing TP712 phage lytic genes. Gene expression was induced at OD_600_ of 0.2 (Time zero) with nisin 2 ng/mL (Nis2) and growth was monitored in a microtiter plate reader. pUK200: empty vector; pUK_AH_H_L: *holTP712-lysTP712*; pLysTP712: l*ysTP712*. For clarity, only the growth curve of *L. lactis* pUK200 without induction is shown because all of the other uninduced clones showed overlapping curves. The results are the average and standard deviations of two independent experiments.

The presence of a putative promoter, which might be activated during the lytic cycle, and the fact that *orf54* and *orf55* (holin) overlap and *orf55* and *orf56* (lysin) are translationally coupled (Figure 1), indicated the putative role of *orf54* in host lysis. *orf54* (GenBank AAX13238.1) encodes a 101-amino acid hypothetical protein with one transmembrane helix domain at its N-terminus. Several gene combinations were cloned in the vector pUK200 and expressed under the inducible nisin promoter in *L. lactis* to assess the role of this *orf54* and the TP712 lysis genes (see Table 2). Attempts to clone the three genes *orf54-holTP712-lysTP712* together failed and only truncated versions of the plasmid were recovered. This result tentatively suggests the high toxicity of the whole phage lytic cassette, because even the basal activity levels of the nisin promoter could not be tolerated. On the other hand, the expression of *holTP712-lysTP712* from the plasmid pUK_AH_H_L (Table 2) seriously compromised growth, once the genes were induced by nisin. After induction, growth rate was initially slowed down and arrested 90 min. after induction (Figure 2). The expression of *lysTP712* alone did not hinder growth. For the first 90 min., the growth of *L. lactis* pLysTP712 was slowed down, but it resumed later on (Figure 2). Therefore, LysTP712 was not able to lyse cells from within and only when co-synthesized with the holin, its production was toxic for the lactococcal cells, albeit without cell lysis, i.e., a drop in OD_600_, occurring.

### 3.2. LysTP712 Activity is Triggered by Membrane Depolarization

**Figure 3:**
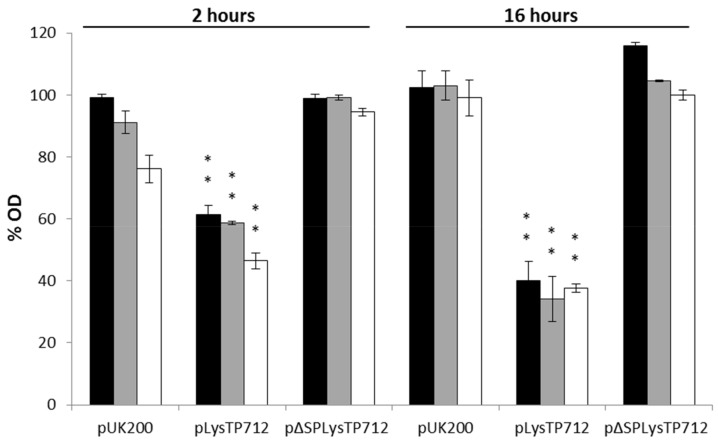
Effect of membrane depolarization on *L. lactis* NZ9000 after synthesis of LysTP712 with or without the signal peptide (pLysTP712 and p∆SPLysTP712, respectively). *L. lactis* NZ9000 cells carrying pLysTP712, pΔSPLysTP712, and pUK200 (empty plasmid) were induced with 2 ng/mL nisin at optical density (OD) of 0.4 and incubated at 30 °C for 2 h. At this time (time zero), cell were treated with 12.5, 25 or 50 ng/mL concentration of nisin (black, grey, and white bars, respectively) or left untreated. OD was measured at 2 and 16 h after nisin exposure. The % OD, relative to non-treated cultures was taken as 100%. Each column represents the means from three independent experiments and error bars indicate the standard deviation. **, *p* < 0.01, significantly different to *L. lactis* pUK200.

We then tested whether membrane-depolarizing agents, mimicking the activity of holins, could trigger lysis in cells expressing *lysTP712* alone, because the expression of the TP712 endolysin gene alone was tolerated. In fact, several ionophores have been shown to sensitize the target cells to the action of several endolysins in both scenarios, when added externally or produced in the cytoplasm [10,11]. In these studies, nisin, a bacteriocin that binds to the cell wall precursor lipid II and forms pores in the cytoplasmic membrane with consequent membrane depolarization [35], was shown to be the most effective ionophore. Therefore, the expression of *lysTP712* was induced in exponentially growing *L. lactis* pLysTP712 and after 2 h cells were challenged with 0, 12.5, 25, or 50 ng/mL (0, 3.75, 7, and 15 nM, respectively) of nisin and the OD_600_ of the cultures was monitored for 16 h. In parallel, the same procedure was applied to *L. lactis* pUK200, carrying the empty plasmid. As shown in Figure 3, the addition of high nisin concentrations slightly inhibited the growth of *L. lactis* pUK200, which was likely due to the high sensitivity of *L. lactis* to nisin, i.e., with minimal inhibitory concentrations within the nanomolar range [36]. The inhibitory effect was significantly more pronounced in *L. lactis* pLysTP712 (*p* < 0.05), but most notably, the growth of *L. lactis* pLysTP712 was not restored after overnight incubation (16 h), while *L. lactis* pUK200 cultures reached the same OD600 as the untreated samples (100%). These results support the hypothesis that membrane permeabilization is required to activate LysTP712 and they are in agreement with the high toxicity observed when *lysTP712* was expressed together with the holin(s) gene. To reinforce this hypothesis further and confirm that growth inhibition was due to LysTP712 activation and not to toxicity that is imposed by too high levels of gene induction after nisin treatment (*lysTP712* was cloned under the inducible nisin promoter), the experiment was also carried out with *L. lactis* pΔSPLysTP712, a 5′ truncated version of *lysTP712,* where the corresponding signal peptide sequence has been deleted (Table 2). Treatment with high nisin doses did not inhibit growth (Figure 3), which suggested that LysTP712 must be translocated to the cell wall first and then become activated after dissipation of the proton motive force.

### 3.3. LysTP712 Is a Sec-Dependent Endolysin

**Figure 4:**
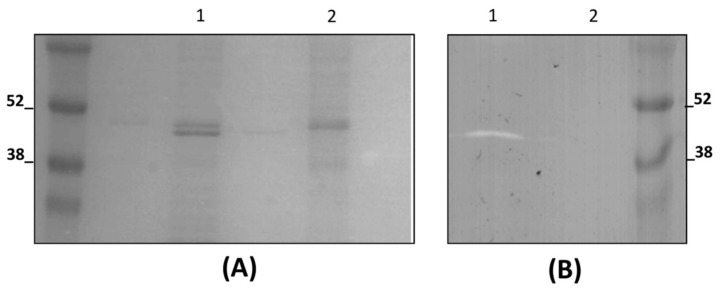
Detection of LysTP712 activity. (**A**) SDS-PAGE analysis of purified His-tagged LysTP712 (Fraction I) expressed in *L. lactis* NZ9000 in the absence (lane 1) or presence (lane 2) of 5 mM sodium azide (NaN_3_). (**B**) Zymogram showing hydrolytic activity of purified LysTP712 expressed in the absence (lane 1) or presence of NaN_3_ (lane 2) in *L. lactis* NZ9000 cells. Faint bands in unlabeled lanes in (**A**) are due to well-to-well overflow.

*lysTP712* codes for a 429 amino acids protein, which is translated into a putative preLysTP712, bearing a signal-peptide-like domain with a predicted signal peptidase I cleavage site between residues A26 and A27, according to the SignalP-5.0 Server. To confirm whether that was the case, *L. lactis* NZ9000/pLysTP712 was induced, and we proceeded to purify LysTP712 with the aid of the C-terminal His tag encoded in pLysTP712. The recombinant lysin was not detected in the supernatant and preliminary attempts to produce and purify it from cell extracts failed, due to the very low solubility of the protein. However, the protein could be recovered under denaturing conditions while using 8 M urea. Recombinant production of LysTP712 in *L. lactis* resulted in a protein band of the expected size for the mature LysTP712 (*ca* 45 kDa) in a 16% SDS-PAGE gel. Its N-terminal sequence (AVGD) confirmed the predicted Sec cleavage site of preLysTP712 after residue A26. However, longer SDS-PAGE runs actually revealed the presence of two proteins bands with similar mobility (Figure 4A, lane 1), which might represent LysTP712 with and without the leader peptide. Only one lytic band was revealed in zymograms that matched with the predicted mature LysTP712 form, i.e., after cleavage of the leader peptide (Figure 4B). To further confirm cleavage of the signal peptide, the widely used Sec inhibitor sodium azide was added just before induction. After purification, only one protein band was recovered, which proved to be inactive in zymograms (Figure 4B, lane 2). Therefore, the LysTP712 precursor form is post-translationally processed in a SecA-dependent manner and the removal of the leader peptide is necessary for the protein to be active.

### 3.4. LysTP712 may Require Additional Components for Activity

**Figure 5:**
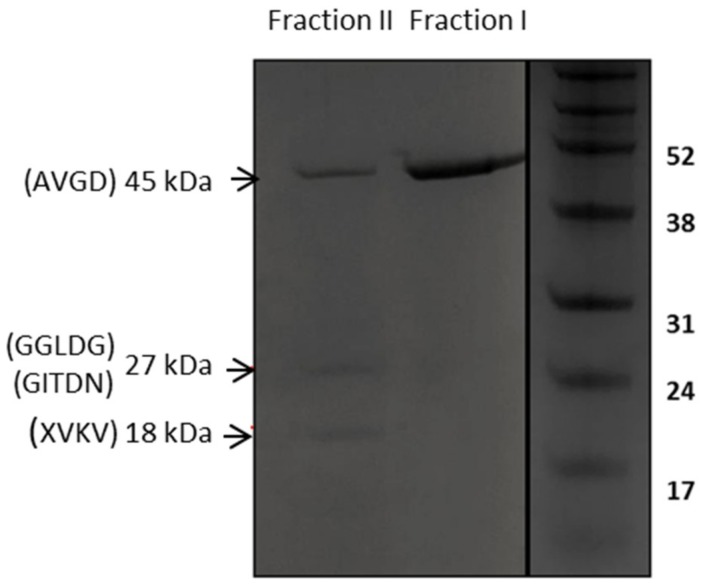
SDS-PAGE analysis of purified His-tagged LysTP712 produced in *L. lactis* NZ9000.

Under our LysTP712 purification protocol, in which elution proceeds at two different pH conditions, the endolysin of the expected molecular weight of ca. 45 kDa was eluted at pH 5.9 (Fraction I), while two polypeptides with apparent molecular weights of 27 and 18 kDa were repeatedly detected in a subsequent fraction at pH 4.5 (Fraction II) (Figure 5). Two N-terminal sequences were obtained from the 27 kDa band, starting at residues G215 (GGLDG) and G225 (GITDN), which are located within the linker immediately after the catalytic domain of LysTP712 that connects both endolysin domains. These extra 27 kDa C-terminal peptides were likely the result of proteolytic degradation, because they were not observed when *lysTP712* was expressed in *L. lactis* PA1001, a strain that lacks the major extracellular protease HtrA [37]. Moreover, neither an ATG start codon nor a putative Shine-Dalgarno sequence was identified.

The proteins were eluted from chromatography column at pH 4.5 (fraction II) and pH 5.9 (fraction I). X, amino acid could not be clearly assigned by N-terminal sequencing.

On the other hand, the 18 kDa band yielded a single N-terminal sequence X_267_VKV (X stands for the first cycle in which the amino acid could not be clearly assigned). This N-terminal sequence is preceded by a Met at position 266 that is located at the N-terminus of the LysTP712 CDB. Inspection of the lysTP712 DNA sequence detected a possible Shine-Dalgarno sequence upstream of the ATG codon (see Figure 1), which anticipated the presence of a secondary translation start site within the gene. This scenario has been described for the so-called multimeric endolysins that required additional copies of the CDBs to increase lysis efficiency [17,18].

To check whether LysTP712 belongs to this endolysin group, we first set out to define an in vitro test to measure its activity when added from the outside. Following production in *L. lactis*, purification and renaturation after dilution in buffer without urea, standard turbidity assays were carried out while using cell suspensions in several buffers and cell lawns on agar plates, but these experiments were not sensitive enough to reveal any lytic activity from any of the two purification fractions (I/II) either alone or in combination. Using target cells from different growth phases (early, mid-exponential, and stationary) and the addition of ionophores, including nigericin and nisin, did not help either. Moreover, no lytic activity was detected after the production and purification from *E. coli* extracts, in which a 5′-truncated version of lysTP712 gene to produce mature LysTP712 (i.e., without the leader peptide) was cloned. In this host, yields were three times higher than in *L. lactis* (1 vs 0.3 mg/mL after purification, respectively) and the putative additional CBD also eluted at pH 4.5, although the synthesis of the 18 kDa protein appears to be more efficient in *L. lactis* (Figure S1).

**Figure 6:**
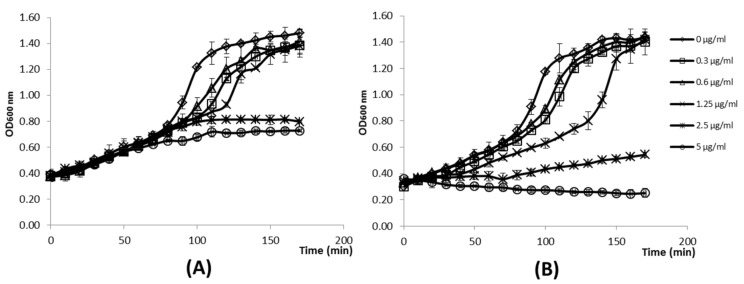
Inhibitory activity of LysTP712 against *L. lactis*. At time point zero, purified LysTP712 from fraction I (**A**) and fraction II (**B**) containing LysTP712FL only, and the endolysin together with the small polypeptides respectively, were added at 0, 0.3, 0.6, 1.25, 2.5 and 5 µg/mL of total protein concentration to early-log-phase cultures of *L. lactis* MG1363. Optical density (OD_600_) of the cultures was monitored in a microtiter plate reader. Plotted data are the means of results from three independent experiments and standard deviations are shown as error bars.

Nevertheless, the activity of LysTP712 could be demonstrated by following growth, once the protein was added to exponentially growing cells, as shown in Figure 6. At time point zero, several concentrations of recombinant LysTP712 from fraction I (Figure 6A) and II (Figure 6B), respectively, were added to mid-log-phase cultures of *L. lactis* (OD_600_, 0.4). The addition of the full length product alone (fraction I) did not affect the growth during the first 90 min. of incubation, although it was arrested later on at LysTP712 concentration of 5 and 2.5 µg/mL (Figure 6A). By contrast, when fraction II was added, the presence of the secondary products seemed to significantly enhance the inhibitory activity (*p* < 0.05) and growth was immediately arrested at the same concentrations and the OD_600_ decreased slightly (Figure 6B). Lower concentrations were ineffective and allowed the cells to recover and reach OD_600_ values that were similar to those of the control sample.

To assess whether additional CBDs could be a limiting factor to LysTP712 activity, a truncated version of lysTP712 starting at the internal translational start site (Met266) was produced in *E. coli* in order to purify the His tagged C-terminal LysTP712 CDB only. Activity assays were then carried out mixing the full length LysTP712 (LysTP712FL) from fraction I with purified CBDs. However, the presence of additional CBD copies did not enhance the inhibitory activity of fraction I. Two different scenarios were tested, as described in Supplementary Figure S2. In one experiment, the cells were preincubated with CBDs at several concentrations and then challenged with LysTP712FL at 0.05 µM (Figure S2A). In another experiment, several molar ratios LysTP712FL::CBD were mixed first and then exponentially added to growing lactococcal cells (Figure S2B). Neither of the approaches improved the activity of LysTP712FL, comparable to that achieved by Fraction II. Thus, although *lysTP712* features an internal translation site and a truncated LysTP712 with the expected N-terminal sequence is produced in homologous and heterologous hosts, the putative role of additional CBDs to enhance its activity could not be confirmed.

### 3.5. The Level of O-Acetylation of L. lactis Peptidoglycan Restricts the Inhibitory Activity of LysTP712

**Figure 7:**
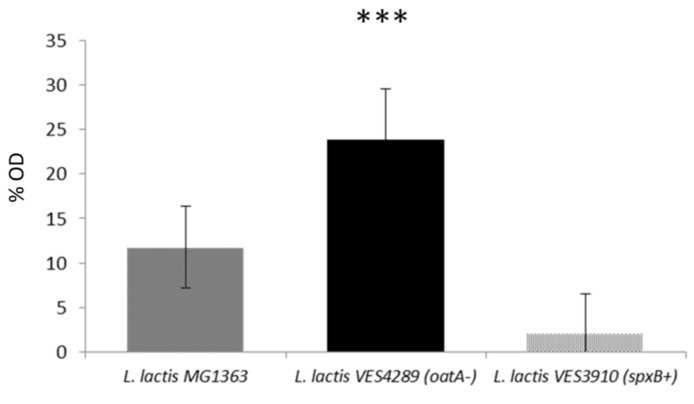
Endolysin LysTP712 sensitivity assay. Early exponential phase cultures (OD_600_, 0.4) of *L. lactis* MG1363, *L. lactis* VES3910 or *L. lactis* VES4289 growing in GM17 medium at 30 °C were treated with 2.5 µg/mL of purified LysTP712 (Fraction II) for 120 min. Columns represent the means of growth inhibition of *L. lactis* relative to untreated cultures ((1 − OD treated/OD untreated) × 100). Bars indicate the standard deviations. ***, *p* < 0,001 significantly different to *L. lactis* MG1363.

According to the CaZy database [38], the catalytic domain of LysTP712 belongs to the glycoside hydrolase family 25 (lysozyme, EC 3.2.1.17), which is responsible for the hydrolysis of (1–4)-β-linkages between *N*-acetyl-muramic acid and *N*-acetyl-D-glucosamine residues. One of the main primary responses of *L. lactis* to lysozyme treatment is the induction of *spxB*, which specifies a RNA polymerase binding regulator that induces the transcription of the PG O-actetylase gene *oatA*, leading to a higher degree of *O*-acetylation of the *N*-acetyl-muramic acid residues in the PG. This change in the composition of the *L. lactis* PG leads to resistance to the catalytic activity of egg white lysozyme [26]. We asked the question of whether such PG modification could inhibit the catalytic activity of LysTP712 and be responsible for, at least partly, the lack of bulk lysis-from-without after addition of LysTP712 to growing lactococcal cells. To this end, *L. lactis* mutants differing in their degree of PG O-acetylation were tested against LysTP712. These strains were *L. lactis* VES3902 overexpressing *spxB*, i.e., with a high degree of *O*-acetylation, and *L. lactis* VES4289, which lacks a functional OatA and, thereby, with un-*O*-acetylated PG [26]. Both mutants significantly differed in their susceptibility to LysTP712 (Figure 7). When added to exponentially growing cells and compared to the wild type strain *L. lactis* MG1363, *L. lactis* VES4289 (*oatA-*) was more sensitive (*p* < 0.001), while the growth of *L. lactis* VES3902 (*spxB+)* was hardly inhibited (Figure 7). Hence, these results support the hypothesis that the degree of PG *O*-acetylation is a factor that impairs the catalytic activity of LysTP712.

## 4. Discussion

The knowledge of key genetic regions that are responsible for the cell burst after the intracellular phage development has generated great interest, which is mostly due to the potential of phage endolysins as promising antimicrobials [39]. In the food arena, lysis of lactococcal cells is also important in the context of flavor formation during the ripening of milk fermented products, such as cheese. Lysis of dairy starter cells is responsible for the release of several peptidases and other intracellular enzymes that participate in the formation of aroma precursors [40]. Indeed, starter cultures have been engineered in the past for the timely production of lactococcal phage endolysins and accelerate cheese ripening [41,42]. Moreover, the recent interest of *L. lactis* as a cell factory makes of programmed lysis a desirable phenotype [43].

The results of this work have demonstrated that the endolysin LysTP712 from the *L. lactis* temperate bacteriophage TP712 requires the disruption of the electrochemical gradient of the plasma membrane to become toxic for the producing cells. On one hand, the co-expression of both the holin and endolysin genes proves toxic to the cell. On the other hand, when *lysTP712* is expressed alone, growth was only halted when cells were sensitized with nisin mimicking the holin pore formation. Lastly, although the use of nisin as both gene inducer as well as ionophore raised questions as to what was halting growth (e.g., toxicity due to uncontrolled gene expression, rather than to activation of LysTP712), it was shown that growth was not arrested after treatment with high nisin doses when the protein was intracellularly produced by the expression of a truncated version of *lysTP712*. As a whole, these results are in agreement with the hypothesis that holin-mediated cell death is required to fully sensitize cells to the lytic action of endolysins that applies to both SAR and Sec-dependent endolysins [2]. Moreover, the putative membrane protein, Orf54, likely plays a part in the putative holin complex, together with the two membrane proteins that are by *holTP712*, given the impossibility of cloning the three genes together. A similar scenario has been described by [9], who were not able to maintain plasmids expressing both the holin and endolysin genes from the *Bacillus* phage SPP1 together.

In line with the prerequisite of the dissipation of the proton motive force to be activated, the results demonstrate that LysTP712 belongs to the so-called exported or secreted endolysins that are endowed with a typical cleavable signal peptide. When produced in *L. lactis*, processing of the N-terminal region was confirmed by protein sequencing. Furthermore, the signal peptide keeps the enzyme inactive and it must be removed, as demonstrated by the lack of lysis in zymograms when *lysTP712* was expressed in the presence of the Sec inhibitor sodium azide. In this way, LysTP712 behaves as Lys44, the first described secretory endolysin from *Oenococcus oeni* phage fOg44 [4]. However, LysTP712 is likely not unique among lactococcal endolysins. Other lactococcal endolysins that are encoded by P335 phages (e.g., AM2, TP901-1, TUC2009, ul36) share a similar architecture and display 83 to 96 % identity with LysTP712, therefore representing a group of secretory endolysin and, probably, a similar activation pathway [14,22,44].

Although not experimentally demonstrated here, it is conceivable that LysTP712 might be a multimeric endolysin that requires the assembly of additional CBDs to achieve an oligomeric conformation for optimal activity, such as the previously identified endolysins Lys170 from the enterococcal phage F170/08 [17] or CTP1L, which lyses *Clostridium tyrobutyricum* [18]. In these examples, the N-terminally truncated C-terminal (CBD) is produced through an internal translation start site within the endolysin gene. In-frame secondary translation has also been reported for the lytic *Staphylococcus aureus* phage 2638A. In this case, the endolysin consists of two enzymatic units and a regulatory or cell wall binding domain, and secondary translation occurs between the gene regions that encode the two enzymatic domains [45]. The presence of an internal start codon at the right location to translate the LysTP712 CBD only and the detection of an 18 kDa protein band with the expected molecular weight and N-terminal sequence is supporting our hypothesis of a similar mode of action for LysTP712. Besides, purification fractions containing both LysTP712 and LysTP712CDB were more active than the full endolysin alone against exponentially growing *L. lactis*. However the activity of LysTP712 was not enhanced by extra CBD units under our experimental conditions, as in the case of CD27L and CS74L endolysins that target *Clostridium difficile* and *Clostridium sporogenes*, respectively [18]. Reconstituting functional multimeric endolysins in vitro is challenging. Indeed, in the case of CPTL1, an excess of CBDs impaired the lysis efficiency due to aggregation or by the saturation of binding sites for the full-length endolysin [18]. Other factors, inherent to the low solubility of the recombinant LysTP712 and improper or incomplete folding after partial urea removal, might also affect its activity negatively. Moreover, the presence of 40 mM urea in the activity assays, while being crucial to avoid protein precipitation, might interfere with proper protein-protein interactions. Therefore, finding other means for keeping the protein soluble should be pursued to provide experimental evidence in support for our hypothesis of a multimeric nature of functional LysTP712.

A clear reduction of cell density was not observed when LysTP712 was produced within the cell (lysis-from-within) and ionophores were added to mimic holin-mediated membrane disruption. It was noticed that, when produced from a plasmid in *L. lactis*, the processing of the signal peptide was not fully achieved and roughly 30% of LysTP712 still retained its signal peptide. In this scenario, it is probable that cell lysis is partially inhibited by the non-active endolysin. On the other hand, attempting to trigger cell lysis like during the lytic phage cycle might not be so straight forward due to the tight and timely regulation of these crucial event for phage survival [2].

Interestingly, our results have shown that the quick response of *L. lactis* to damage of its peptidoglycan might restrain the activity of the endolysin in growing cells. The response of *L. lactis* to cell envelope damage is orchestrated by the two component system CesSR, which immediately induces several genes that are involved in the modification of its cell wall [26,46]. One of the members of the CesSR regulon is *spxB*, which in turn binds to RNA polymerase and directs the transcription of *oatA*, encoding the PG O-acetylase OatA [26]. The main consequence of this activation cascade is the higher degree of O-acetylation of the muropeptides, leading to the resistance to lysozyme, precisely the predicted catalytic activity of LysTP712. Our results have shown that LysTP712 is indeed sensitive to PG O-acetylation, because *L. lactis oatA*- was more sensitive than the wild-type strain, and a mutant with a higher degree of PG *O*-acetylation was hardly inhibited in our LysTP712 activity assay. Although resistance to phage endolysins is rare because they are fast-acting enzymes, leaving the target cells with no chance to respond, there are some reports of resistance to other PG hydrolases, such as autolysins and egg-white lysozyme, which belong to the same glycoside hydrolase family as LysTP712, in lactococci and in other Gram positive bacteria [47,48,49,50]. Moreover, this is not the first case describing how the physiology of the target cells has a negative impact on the activity of phage endolysins. For example, the lytic activity of LysSPP1 from the *Bacillus* phage SPP1 was compromised when the *Bacillus* cells were grown under aeration conditions [9].

In summary, the lytic function of the temperate phage TP712 relies on the expression of holin proteins and the LysTP712 endolysin, which requires membrane depolarization to be activated. Moreover, LysTP712 belongs to the growing family of secretory endolysins and likely requires an oligomeric state in complexes with additional CBDs for full activity. Finally, LysTP712 is sensitive to *O*-acetylation of the lactococcal peptidoglycan, which demonstrates that this modification inhibits not only host autolysins but also phage endolysins.

## Figures and Tables

**Table 1:** Bacterial strains used in this work.

Strain	Description	Reference
*E. coli* DH10B	*E. coli* cloning host	Invitrogen
*E. coli* BL21 (pLys)	Gene expression host	Novagen
*L. lactis* MG1363	Plasmid-free derivative of NCDO712	[24]
*L. lactis* NZ9000	MG1363 *pepN*::*nisRK*. Host for nisin inducible gene expression	[25]
*L. lactis* UKLc10 TP712	TP712 lysogen and host for nisin inducible expression	[21]
*L. lactis* VES3910	MG1363 carrying pVES3910 (*spxB*+)	[26]
*L. lactis* VES4289	MG1363 lacking a functional *oatA* (*oatA-*)	[26]

**Table 2:** Plasmids used in this study.

Plasmid	Description *	Reference
pUK200	Nisin inducible *L. lactis* expression vector. CmR.	[27]
pET21a	IPTG inducible *E. coli* expression vector. AmpR.	EMD Biosciences
pUK_AH_H_L	*holTP712-lysTP712* under the nisin inducible promoter in pUK200.	This work
pLysTP712	*lysTP712*-His6 under the nisin inducible promoter in pUK200.	This work
pΔSPLysTP712	leaderless LysTP712 under the nisin inducible promoter in pUK200.	This work
pETLysTP712	pET21a::ΔSP*lysTP712*-His6.	This work
pETLysTP712CBD	pET21a::lysTP712CBD-His6.	This work

* CmR, Chloramphenicol resistance; AmpR, Ampicillin resistance; ΔSP*lysTP712*-His6, truncated *lysTP712* gene encoding leaderless LysTP712 and fused to a 6x His tag; lysTP712CBD-His6, LysTP712 cell binding domain fused to a 6× His tag.

## Supplementary Materials

The following are available online at https://www.mdpi.com/1999-4915/11/10/881/s1, Figure S1: SDS-PAGE analysis of purified His-tagged LysTP712, Figure S2: Inhibitory activity of LysTP712 against *L. lactis* in the presence of additional cell binding domains.
