# Supplementary material for: Insight into the Lytic Functions of the Lactococcal Prophage TP712
Source: Viruses. 2019 Sep 20;11(10):881. doi: 10.3390/v11100881 (PMC6832245; doi:10.3390/v11100881)
Supplement: Supplementary file 1 [file viruses-11-00881-s001.zip › Supp Fig 1 Rev.pdf]

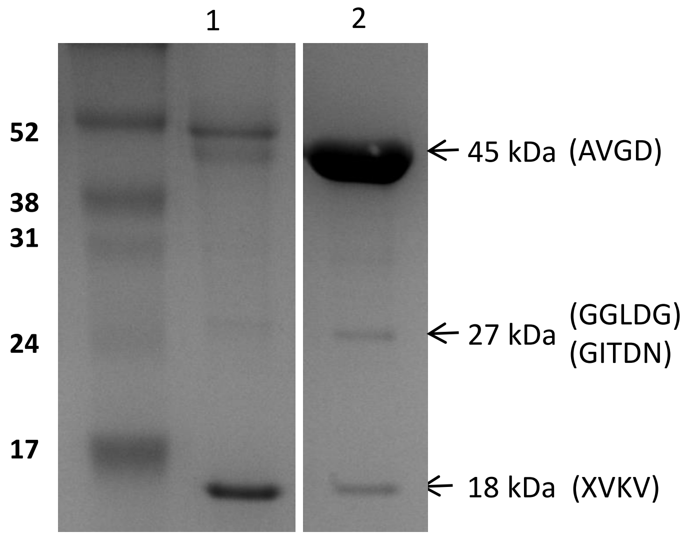

Supplementary Figure 1. SDS-PAGE of recombinant LysTP712 produced by *L. lactis* NZ9000/pLysTP712 (Lane 1) and *E. coli* BL21 (pLys)/pETLysTP712 (Lane 2) eluted at pH 4.5 (Fraction II). Total protein loaded in the gel was 1.8 µg and 5.4 µg, respectively.
