# Supplementary material for: Insight into the Lytic Functions of the Lactococcal Prophage TP712
Source: Viruses. 2019 Sep 20;11(10):881. doi: 10.3390/v11100881 (PMC6832245; doi:10.3390/v11100881)
Supplement: Supplementary file 1 [file viruses-11-00881-s001.zip › Supp Fig 2 Rev.pdf]

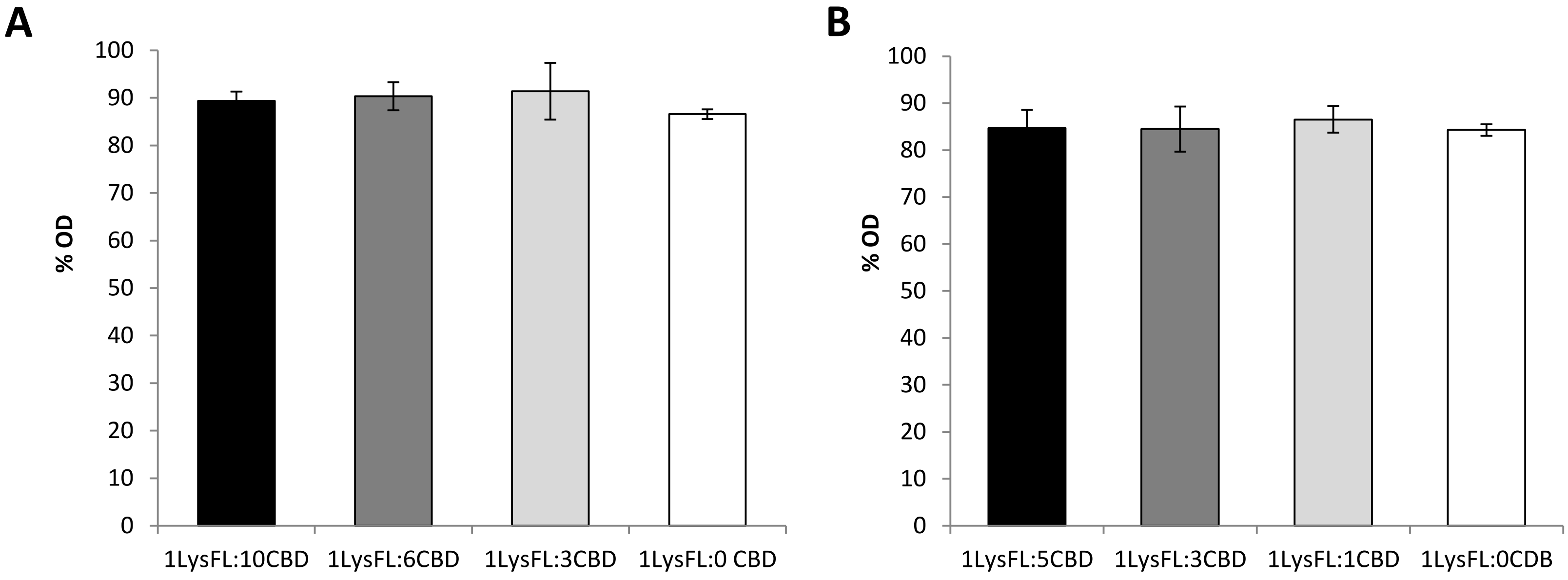

Supplementary Figure 2. Inhibitory activity of LysTP712 against *L. lactis* in the presence of additional cell binding domains (CBD). (A) Mid log cultures of *L. lactis* MG1363 were preincubated with LysTP712CDB and treated with LysTP712 (0.05 µM) to get molar ratios LysTP712:CBD of 1:10, 1:6, 1:3 or without CBDs (black, dark grey, light grey and white bars, respectively). (B) LysTP712FL at 0.05 µM was co-incubated first with LysTP712CDB at 1:1, 1:3 and 1:5 molar ratios or without CBDs before addition to mid log cultures of *L. lactis* MG1363 (black, dark grey, light grey and white bars, respectively). Columns represent growth of treated cultures relative to untreated cultures (taken as 100%) after 120 min. Means and standard deviations of three independent experiments are represented.
